# Comprehensive analyses of the microRNA–messenger RNA–transcription factor regulatory network in mouse and human renal fibrosis

**DOI:** 10.3389/fgene.2022.925097

**Published:** 2022-11-15

**Authors:** Le Deng, Gaosi Xu, Qipeng Huang

**Affiliations:** ^1^ Department of Nephrology, The Second Affiliated Hospital of Nanchang University, Jiangxi, China; ^2^ Department of Nephrology, The Fifth Affiliated Hospital of Jinan University, Heyuan, China

**Keywords:** therapeutic targets, miRNA–mRNA–transcription factor regulatory network, unilateral ureteral obstruction, renal fibrosis, bioinformatics analysis

## Abstract

**Objective:** The aim of this study was to construct a microRNA (miRNA)–messenger RNA (mRNA)–transcription factor (TF) regulatory network and explore underlying molecular mechanisms, effective biomarkers, and drugs in renal fibrosis (RF).

**Methods:** A total of six datasets were downloaded from Gene Expression Omnibus. “Limma” and “DESeq2” packages in R software and GEO2R were applied to identify the differentially expressed miRNAs and mRNAs (DEmiRNAs and DEmRNAs, respectively). The determination and verification of DEmiRNAs and DEmRNAs were performed through the integrated analysis of datasets from five mouse 7 days of unilateral ureteral obstruction datasets and one human chronic kidney disease dataset and the Human Protein Atlas (http://www.proteinatlas.org). Target mRNAs of DEmiRNAs and TFs were predicted by prediction databases and the iRegulon plugin in Cytoscape, respectively. A protein–protein interaction network was constructed using STRING, Cytoscape v3.9.1, and CytoNCA. Functional enrichment analysis was performed by DIANA-miRPath v3.0 and R package “clusterProfiler.” A miRNA–mRNA–TF network was established using Cytoscape. Receiver operating characteristic (ROC) curve analysis was used to examine the diagnostic value of the key hub genes. Finally, the Comparative Toxicogenomics Database and Drug-Gene Interaction database were applied to identify potential drugs.

**Results:** Here, 4 DEmiRNAs and 11 hub genes were determined and confirmed in five mouse datasets, of which *Bckdha* and *Vegfa* were further verified in one human dataset and HPA, respectively. Moreover, *Bckdha* and *Vegfa* were also predicted by miR-125a-3p and miR-199a-5p, respectively, in humans as in mice. The sequences of miR-125a-3p and miR-199a-5p in mice were identical to those in humans. A total of 6 TFs were predicted to regulate *Bckdha* and *Vegfa* across mice and humans; then, a miRNA–mRNA–TF regulatory network was built. Subsequently, ROC curve analysis showed that the area under the curve value of *Vegfa* was 0.825 (*p* = 0.002). Finally, enalapril was identified to target *Vegfa* for RF therapy.

**Conclusion:** Pax2, Pax5, Sp1, Sp2, Sp3, and Sp4 together with *Bckdha*-dependent miR-125a-3p/*Vegfa*-dependent miR-199a-5p formed a co-regulatory network enabling *Bckdha*/*Vegfa* to be tightly controlled in the underlying pathogenesis of RF across mice and humans. *Vegfa* could act as a potential novel diagnostic marker and might be targeted by enalapril for RF therapy.

## Introduction

Renal fibrosis (RF) is a final common pathway of many forms of kidney disease and ultimately leads to chronic kidney disease (CKD) progression and end-stage kidney disease (Liu and Zhuang, 2019). RF is commonly accepted as the key to regulating the progression of CKD (Farris and Alpers, 2011). Therefore, it is of great significance to explore the potential pathogenesis of RF (Chen et al., 2019).

Over the past decade, a growing body of experimental work has demonstrated that the expression of microRNAs (miRNAs) and their downstream target genes are closely connected with RF (Liu Y. et al., 2019; Fu et al., 2020), and suggests that the possibility of RF prevention *via* miRNA regulation targeting related genes may contribute to novel treatment for RF (Liu L. et al., 2019). In addition, transcription factors (TFs) widely participate in the process of RF through the regulation of specific gene expression and play a critical role in RF (Miranda et al., 2021; Li et al., 2021).

The miRNA–mRNA–TF network plays a crucial role in the regulatory mechanism of RF. Previous research studies have often focused on the role of a single molecule in RF while neglecting the crucial synergistic effects of miRNAs and TFs in the context of genetic regulatory networks and the deeper molecular mechanism in RF. Moreover, the regulatory networks are still largely ambiguous. Unilateral ureteral obstruction (UUO) is a classical and widely applied experimental animal model for researching RF (Humphreys, 2018; Martínez-Klimova et al., 2019). At 7 days of UUO (7dUUO), interstitial fibrosis was already detected accompanied by blood vessel collapse, inflammatory cell infiltration, tubular atrophy, and enhanced extracellular matrix production (Sun et al., 2013). Currently, the diagnostic biomarkers and therapeutic drugs for RF remain limited. In the present study, we performed an integrated analysis of five mouse 7dUUO datasets and one human CKD dataset and constructed a comprehensive miRNA–mRNA–TF regulatory network across mice and humans. In addition, the receiver operating characteristic (ROC) curve analysis was applied to assess the diagnostic value of the key hub genes. Moreover, possible specific therapeutic drugs targeting the key hub genes were identified, which may provide a novel therapeutic direction for RF.

## Materials and methods

### Data collection

**FIGURE 1:**
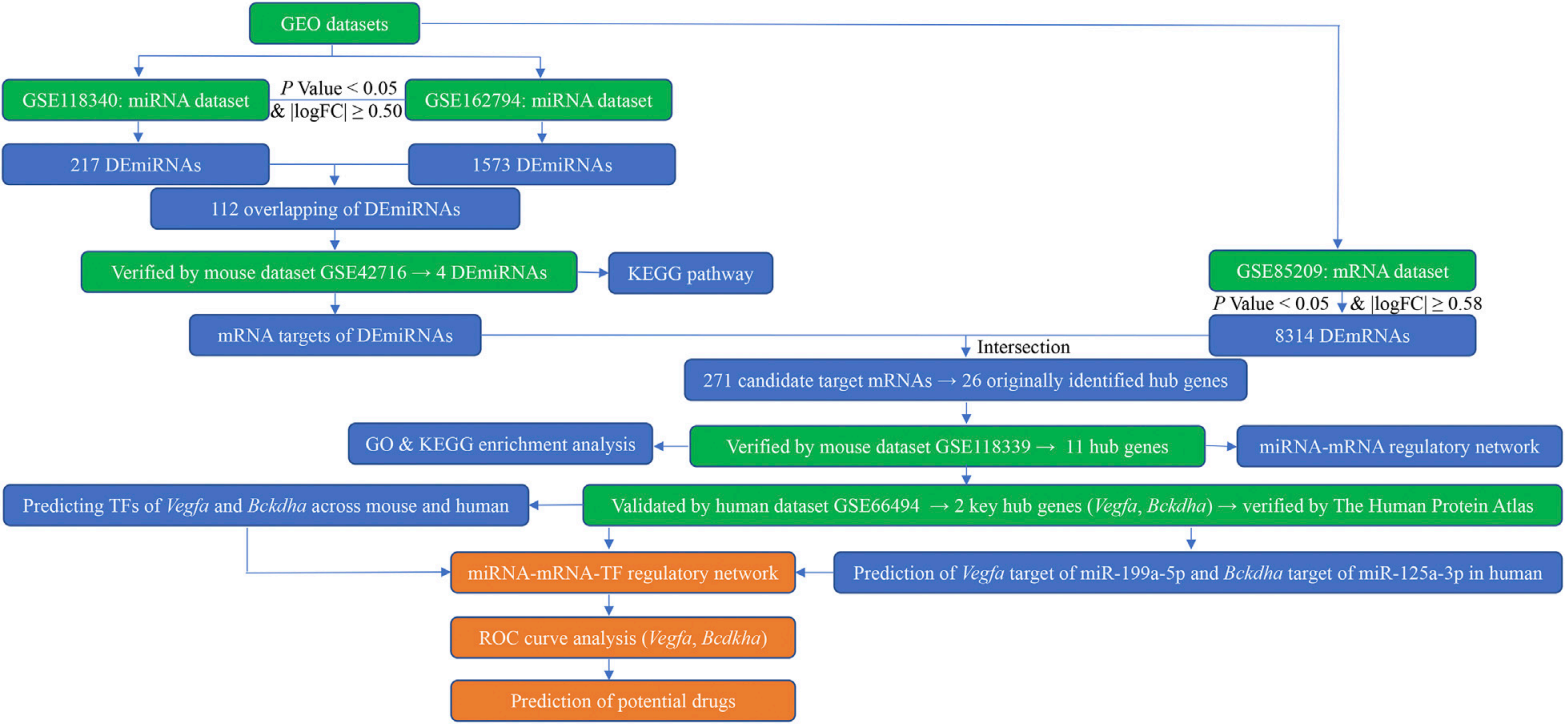
Flow diagram of bioinformatics analysis. GEO, Gene Expression Omnibus; miRNA, microRNA; mRNA, messenger RNA; logFC, log fold change; DEmiRNAs, differentially expressed miRNAs; DEmRNAs, differentially expressed mRNAs; KEGG, Kyoto Encyclopedia of Genes and Genomes; GO, Gene Ontology; TFs, transcription factors; ROC, receiver operating characteristic.

Three high-throughput datasets (GSE118340, GSE85209, and GSE118339) and three microarray datasets (GSE162794, GSE42716, and GSE66494) were downloaded from the National Center for Biotechnology Information (NCBI) Gene Expression Omnibus (GEO), respectively (https://www.ncbi.nlm.nih.gov/geo/) (December 2021). The mouse renal samples were harvested from *Mus musculus* 7 days after UUO surgery and then were subjected to analysis of miRNAs and mRNAs. Each mouse dataset was divided into a UUO (7dUUO) group and without UUO control group. Human dataset GSE66494 contains two independent sets: a discovery set including 47 biopsy specimens of CKD patients and 5 normal kidney samples and a validation set consisting of 5 biopsy specimens of CKD patients and 3 normal kidney samples, of which, the validation set was utilized as a human validation dataset in the present study and was divided into a CKD group and normal group. The flow diagram of the study design is shown in Figure 1.

### Screening differentially expressed miRNAs and differentially expressed mRNAs

DEmiRNAs of the GSE162794 and GSE42716 datasets between the UUO group and control group were identified using GEO2R (http://www.ncbi.nlm.nih.gov/geo/geo2r/). Raw data of GSE118340, GSE85209, and GSE118339 datasets were extracted from the GEO. DEmiRNAs of the GSE118340 dataset and DEmRNAs of the GSE85209 and GSE118339 datasets between the UUO group and control group were identified using the R software “limma” and “DESeq2” packages, and principal component analysis (PCA) of the three datasets was performed by using R software “FactoMineR” and “factoextra” packages. The criteria of DEmiRNAs were |log fold change (FC) | ≥ 0.50 and *p-*value <0.05, and the cutoff values of DEmRNAs were set as *p-*value <0.05 and |logFC| ≥ 0.58. The visualization of DEmiRNAs and DEmRNAs was realized in the volcano plot and heatmap *via* “ggplot2” and “ComplexHeatmap” packages, respectively. Then, the “VennDiagram” package was applied to identify the intersection of DEmiRNAs in the GSE118340 and GSE162794 datasets, and consistently changed DEmiRNAs were extracted as candidate DEmiRNAs for further analysis.

### Determination and validation of screening differentially expressed miRNAs

Using the same screening criteria (*p-*value <0.05 and |logFC| ≥ 0.50), the GSE42716 dataset was used to determine and validate the candidate DEmiRNAs. The “VennDiagram” package was employed to identify the overlaps of DEmiRNAs in GSE42716 and the candidate DEmiRNAs, and then consistently changed DEmiRNAs were selected and their expression levels were further verified between the UUO group and control group in the GSE42716 dataset.

### Prediction of target mRNAs of screening differentially expressed miRNAs and acquisition of candidate target mRNAs

MiRDB (Chen and Wang, 2020) (http://www.mirdb.org), TargetScan (McGeary et al., 2019) (http://www.targetscan.org/vert_80/), miRWalk (Sticht et al., 2018) (http://mirwalk.umm.uni-heidelberg.de/), DIANA-microT-CDS (Paraskevopoulou et al., 2013) (http://www.microrna.gr/microT-CDS), and starBase (Li et al., 2014) (http://starbase.sysu.edu.cn) (February 2022) were applied to search the predicted target mRNAs of DEmiRNAs. We chose the intersection of at least three of the five databases by the “Draw Venn Diagram” online tool (bioinformatics.psb.ugent.be/webtools/Venn), and then by matching the DEmRNAs selected from the GSE85209 dataset using the R software “VennDiagram” package, the overlapping mRNAs were screened as the candidate target mRNAs.

### Construction of the protein–protein interaction network and validation of originally identified hub genes in the mouse dataset

The selected candidate target mRNAs were uploaded to the protein–protein interaction (PPI) network. The PPI network was constructed with the threshold of the minimum required interaction score >0.4 using the Search Tool for the Retrieval of Interacting Genes/Proteins (STRING) database (Szklarczyk et al., 2021) (version:11.5; http://cn.string-db.org) and Cytoscape software (Otasek et al., 2019) (v3.9.1; https://cytoscape.org) (February 2022). Subsequently, the Cytoscape plugin–CytoNCA was utilized to calculate the scores of gene nodes by applying three centrality methods (degree centrality, betweenness centrality, and closeness centrality). High degree centrality (DC) is at the network center and is considered a crucial research focus. A node degree >5 was regarded as the criterion for screening originally identified hub genes. Then, another independent mouse dataset, GSE118339, was utilized to verify the expression level of the originally identified hub genes between the UUO group and control group, and then hub genes were obtained.

### Construction of the miRNA–mRNA regulatory network and functional and pathway enrichment analysis

Based on the results of verified DEmiRNAs and hub genes, the miRNA–mRNA regulatory network in mice was built and visualized by using Cytoscape. To further explore the major biological functions of these DEmiRNAs and hub genes, Kyoto Encyclopedia of Genes and Genomes (KEGG) pathway analysis for the validated DEmiRNAs was undertaken by DIANA-miRPath v3.0 (Vlachos et al., 2015) (February 2022) and R package “clusterProfiler” from Bioconductor was used to perform Gene Ontology (GO) and KEGG pathway analyses for the hub genes. GO term enrichment analysis mainly contains a biological process (BP), cellular component (CC), and molecular function (MF). A *p-*value <0.05 was considered statistically significant. The significant items of GO were visualized using the “ggplot2” package. A hub gene–KEGG interaction network was built using Cytoscape.

### Verification of hub genes in humans and construction of the miRNA–mRNA–TF regulatory network

To validate the clinical value of the results, a human dataset GSE66494 was applied to focus on the expression of hub genes between biopsy specimens of CKD patients and normal kidney samples, and then key hub genes were identified. In addition, the Human Protein Atlas, available online (http://www.proteinatlas.org) (February 2022), was used to further verify the expression of key hub genes (Thul et al., 2017). Next, to explore whether the key hub genes could also be predicted by their paired miRNAs in humans as in mice, miRDB, TargetScan, miRWalk, DIANA-microT-CDS, and starBase databases (February 2022) were again employed. Next, to examine the conservation of miRNAs between mice and humans, the sequence analysis of the DEmiRNAs associated with the key hub genes were conducted using miRBase (http://www.mirbase.org) (July 2022) (Kozomara et al., 2019).

Potential TFs associated with key hub genes were predicted by using the iRegulon plugin in Cytoscape across mice and humans (Janky et al., 2014) with all the default parameters. Transcription factors co-predicted both in mice and humans and TF motifs with a normalized enrichment score (NES) > 5 in Cytoscape were identified as the thresholds for the selection of potential associations. Then, a miRNA–mRNA–TF regulatory network across mice and humans was built and visualized using Cytoscape software. In addition, ROC curve analysis was conducted to evaluate the diagnostic value of key hub genes in the validation set of the GSE66494 dataset.

### Prediction of potential drugs

The Comparative Toxicogenomics Database (CTD; http://ctdbase.org/) (February 2022) is a digital ecosystem database, which involves toxicological information for genes, phenotypes, chemicals, and diseases (Davis et al., 2021). We appointed the disease as “Nephrosclerosis” and identified chemicals related to key hub genes. In addition, key hub genes were also queried using the Drug-Gene Interaction database (DGIdb), a gene–drug interaction online tool based on the combination of expert curation and text mining (February 2022) (Cotto et al., 2018). The overlaps of the CTD and DGIdb were selected as the potential drugs.

### Statistical analysis

Data were analyzed with statistical product and service solutions (SPSS) statistical software for Windows, version 24.0 (SPSS Inc., Chicago, Illinois, United States), and the R statistical software package (version 4.1.2). The one-sample Shapiro–Wilk test was used to detect whether variables were of normal distribution. The expression levels of DEmiRNAs and mRNAs targeted by DEmiRNAs between the two comparison groups were validated by the Mann–Whitney *U* test (skewed distribution continuous variables) and Student’s *t*-test (normal distribution continuous variables) and visualized using GraphPad Prism 7.00. ROC curves of key hub genes were plotted using the “pROC” package.

## Results

### Expression profiles of miRNA and mRNA

The miRNA expression profiles were from three datasets, namely, GSE118340, GSE162794, and GSE42716. The mRNA expression profiles were from GSE85209, GSE118339, and GSE66494 datasets. There were 23 samples in the miRNA expression profiles, i.e., 12 7dUUO kidney samples and 11 without UUO kidney samples. There were 22 samples in mRNA expression profiles, i.e., 14 mouse kidney samples (8 7dUUO kidney tissues and 6 without UUO kidney tissues) and 8 human kidney samples (5 biopsy specimens of CKD patients and 3 normal kidney samples). The basic information of the six datasets is given in Table 1.

**TABLE 1:** Basic information of six datasets.

GEO ID	Platform	Author	Year	Country	Tissue	Number of cases (7dUUO vs. without UUO or CKD vs. normal)
microRNA expression profiling						
GSE118340	GPL19057	Pantano L, Pavkovic M	2019	United States	Kidney (*Mus musculus*)	4 vs. 3
GSE162794	GPL21265	Kaneko S	2021	Japan	Kidney (*Mus musculus*)	4 vs. 4
GSE42716	GPL10384	Morizane R, Monkawa T	2015	Japan	Kidney (*Mus musculus*)	4 vs. 4
mRNA expression profiling						
GSE118339	GPL19057	Pantano L, Pavkovic M	2019	United States	Kidney (*Mus musculus*)	4 vs. 3
GSE85209	GPL19057	Gerarduzzi C, Vaidya VS., Hutchinson JN	2019	United States	Kidney (*Mus musculus*)	4 (WT) vs. 3 (WT)
GSE66494	GPL6480	Masuda S, Nakagawa S, Nishihara K, *etc.*	2019	Japan	Kidney (*Homo sapiens*)	5 vs. 3 (validation set)

GEO, Gene Expression Omnibus; UUO, unilateral ureteral obstruction; CKD, chronic kidney disease; miRNA, microRNA; vs., *versus*; mRNA, messenger RNA; WT, wild type.

### Identification of differentially expressed miRNAs and differentially expressed mRNAs

**FIGURE 2:**
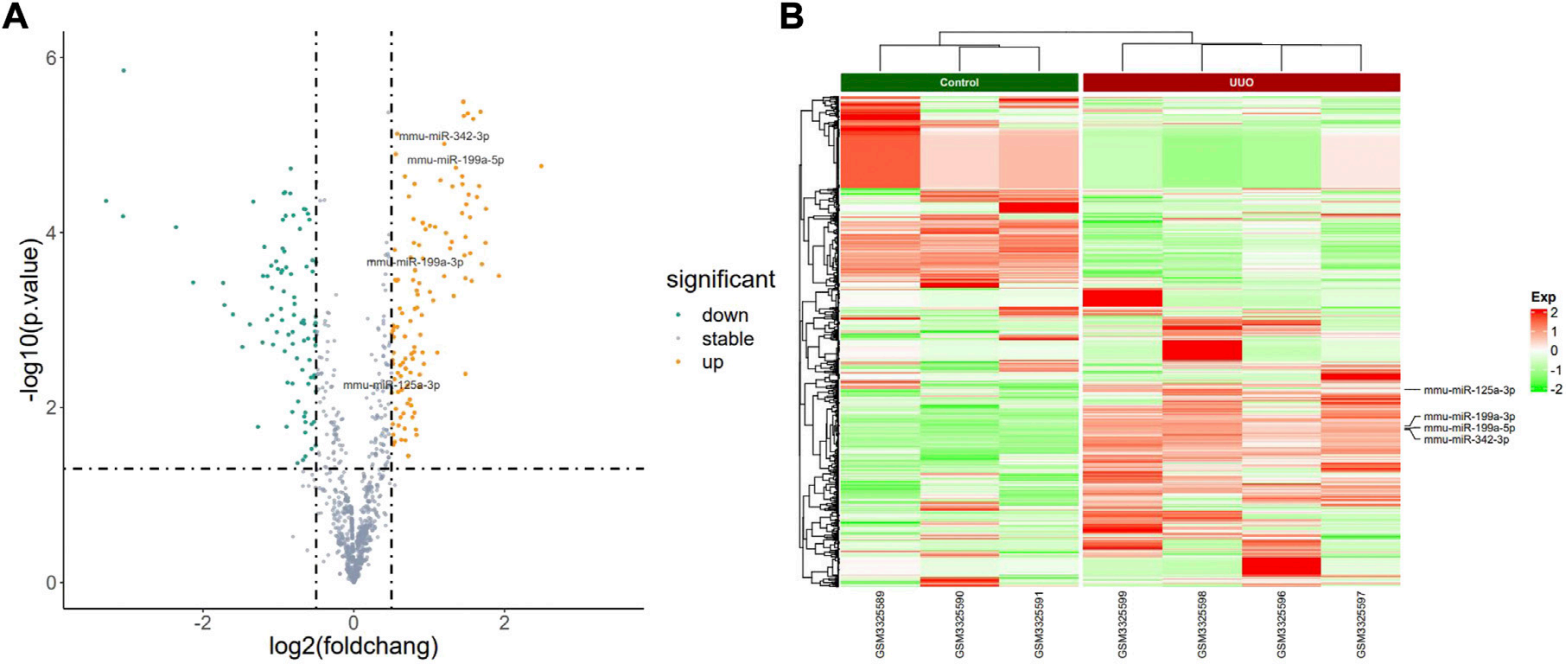
Profiling of DEmiRNAs in the UUO group and control group in GSE118340. **(A)** Volcano plots. Orange represents upregulation and green represents downregulation. **(B)** Heatmaps. Red represents upregulation and green represents downregulation.

**FIGURE 3:**
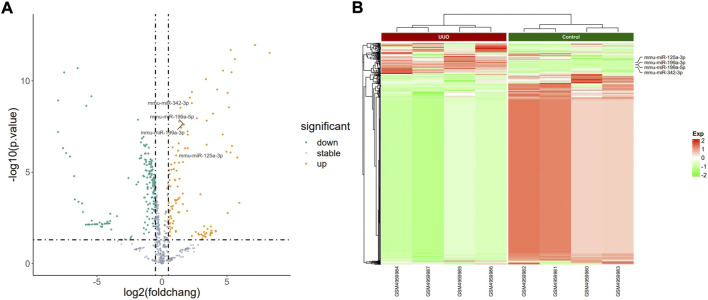
Profiling of DEmiRNAs in the UUO group and control group in GSE162794. **(A)** Volcano plots. Orange represents upregulation and green represents downregulation. **(B)** Heatmaps. Red represents upregulation and green represents downregulation.

**FIGURE 4:**
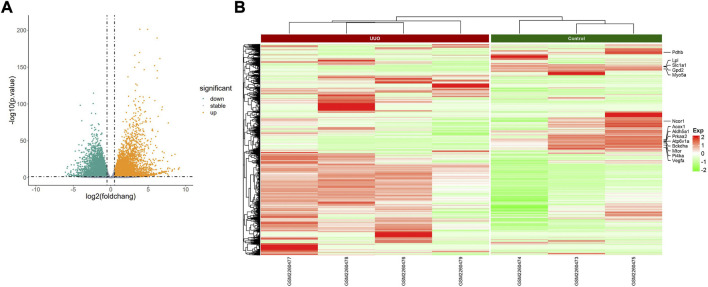
Profiling of DEmRNAs in the UUO group and control group in GSE85209. **(A)** Volcano plots. Orange represents upregulation and green represents downregulation. **(B)** Heatmaps. Red represents upregulation and green represents downregulation.

The PCA plot showed the tight clustering of biological replicates and distinct clustering between the UUO group and control group in the GSE118340, GSE85209, and GSE118339 datasets, and the data could be used for further analysis (Supplementary Figure S1). Based on the screening criteria, there were 217 and 1,573 DEmiRNAs in GSE118340 (126 upregulation and 91 downregulation) and GSE162794 (112 upregulation and 1,461 downregulation), respectively, and 112 DEmiRNAs (35 upregulation and 77 downregulation) were consistently changed in the two datasets, which were regarded as originally identified DEmiRNAs (Supplementary Figure S2A). In addition, in the GSE85209 dataset, a total of 8,314 DEmRNAs were detected, which consisted of 4,436 upregulated and 3,878 downregulated DEmRNAs. The distribution of differential miRNA and mRNA expression in the UUO group and control group of the GSE118340, GSE162794, and GSE85209 datasets was intuitively shown by the volcano plot and heatmap (Figures 2–4).

### Determination and validation of differentially expressed miRNAs

**FIGURE 5:**
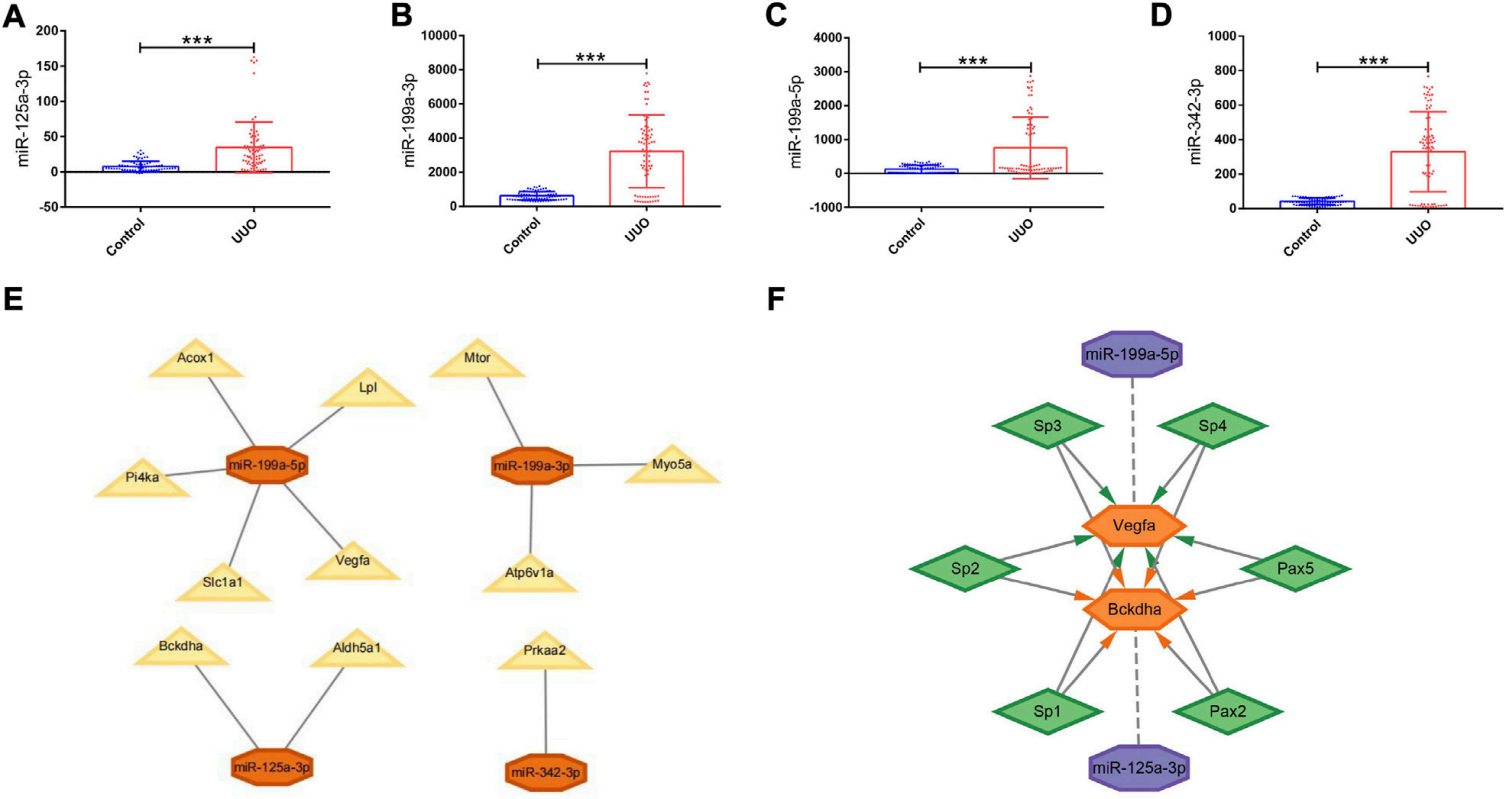
Expression levels of DEmiRNAs in the UUO group and control group, PPI network, and regulatory networks of the miRNA–mRNA and miRNA–mRNA–TF. **(A–D)** Expression levels of DEmiRNAs in the UUO group and control group based on the GSE42716 dataset. **(A)** miR-125a-3p. **(B)** miR-199a-3p. **(C)** miR-199a-5p. **(D)** miR-342-3p. **(E)** MiRNA–mRNA regulatory network. Orange octagons represent miRNAs and yellow triangles represent mRNAs. **(F)** MiRNA–mRNA–TF regulatory network. Purple octagons indicate miRNAs, orange hexagons indicate mRNAs, and green diamonds indicate TFs. DEmiRNAs, differentially expressed miRNAs; UUO, unilateral ureteral obstruction; TF, transcription factor. *p < 0.05; **p < 0.01; and ***p < 0.001.

GSE42716 was employed as one mouse validation dataset. A total of 174 DEmiRNAs were detected, among which 5 were downregulated and 169 were upregulated based on the same screening standard (*p-*value <0.05 and |logFC| ≥ 0.50). Among the 112 originally identified DEmiRNAs, four DEmiRNAs were detected to be upregulated simultaneously in the GSE42716 dataset, while no downregulated DEmiRNA was observed (Supplementary Figure S2B). Then, we focused on the expression levels of the four DEmiRNAs, namely, miR-125a-3p, miR-199a-3p, miR-199a-5p, and miR-342-3p, which exhibited significant expression differences between the UUO group and control group in the GSE42716 dataset (Figures 5A–D).

### Prediction of target mRNAs for differentially expressed miRNAs and acquisition of candidate target mRNAs

Here, 455 targets for miR-125a-3p, 478 targets for miR-199a-3p, 490 targets for miR-199a-5p, and 458 targets for miR-342-3p were predicted in at least three of the five databases (Supplementary Figure S3). No result matched “miR-125a-3p” in the starBase database. Finally, a total of 1,695 target mRNAs were predicted to bind the four DEmiRNAs. Given the fact that target mRNAs are generally negatively regulated by miRNAs, by taking the intersection of the 1,695 predicted target mRNAs and 3,878 downregulated DEmRNAs obtained from the GSE85209 dataset by the R software “VennDiagram” package, in total, 271 downregulated target mRNAs were selected as candidate target mRNAs (Supplementary Figure S4).

### Construction of the PPI network and validation of originally identified hub genes

The 271 proteins encoded by candidate target mRNAs were searched in the STRING database and then the PPI network was built, which included 212 nodes and 304 pairs of edges (Supplementary Figure S5). In addition, with the criterion of node degree >5, 26 originally identified hub genes were identified using the cytoNCA plugin for further analysis (Supplementary Table S1).

**FIGURE 6:**
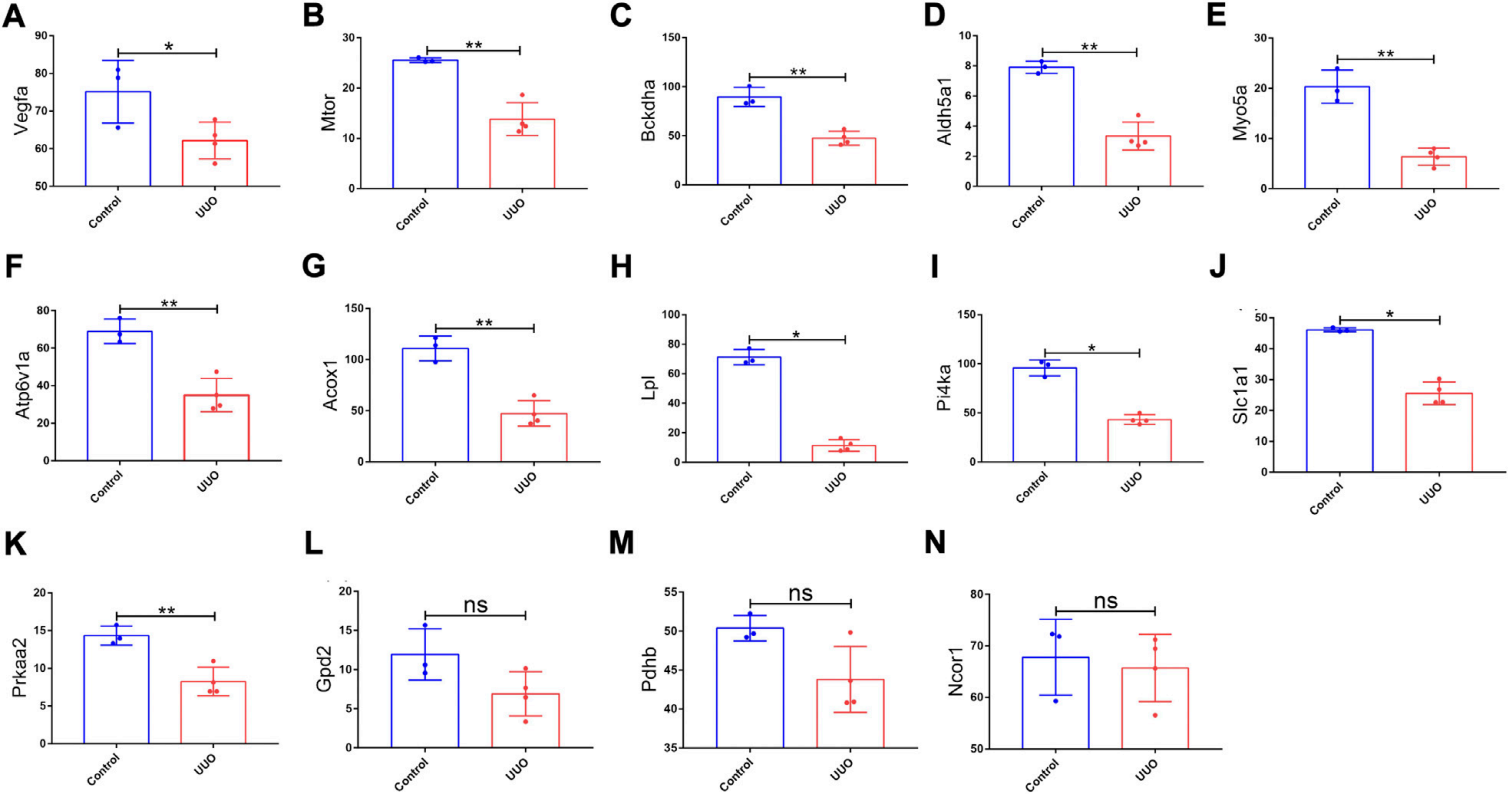
Expression levels of 14 genes in the UUO group and control group based on the GSE118339 dataset. The 14 genes were from 26 originally identified hub genes, which were also detected by the GSE118339 dataset. **(A)** *Vegfa*; **(B)** *Mtor*; **(C)** *Bckdha*; **(D)** *Aldh5a1*; **(E)** *Myo5a*; **(F)** *Atp6v1a*; **(G)** *Acox1*; **(H)** *Lpl*; **(I)** *Pi4ka*; **(J)** *Slc1a1*; **(K)** *Prkaa2*; **(L)** *Gpd2*; **(M)** *Pdhb*; and **(N)** *Ncor1*. UUO, unilateral ureteral obstruction; ns, no significance; *p < 0.05 and **p < 0.01.

GSE118339 was utilized as the other mouse validation dataset. According to the same screening standard (*p-*value <0.05 and |logFC| ≥ 0.58), 1,102 upregulated and 1,460 downregulated DEmRNAs were detected. Among the 26 originally identified hub genes, 14 genes were detected in the GSE118339 dataset. Of the 14 genes, 11 showed the same change direction as the GSE118339 dataset and exhibited significant differences and were regarded as hub genes (Figure 6).

### Construction of the miRNA–mRNA regulatory network in mice and functional and pathway enrichment analysis

Derived from the analysis results of hub genes and validated DEmiRNAs, a miRNA–mRNA regulatory network in mice was established by using Cytoscape, involving 4 DEmiRNAs and 11 hub genes (Figure 5E). In such a regulatory network, miR-125a-3p targets *Bckdha* and *Aldh5a1*; miR-199a-3p targets *Mtor*, *Myo5a*, and *Atp6v1a*; miR-199a-5p targets *Vegfa*, *Acox1*, *Lpl*, *Pi4ka*, and *Slc1a1*; and miR-342-3p targets *Prkaa2*.

**FIGURE 7:**
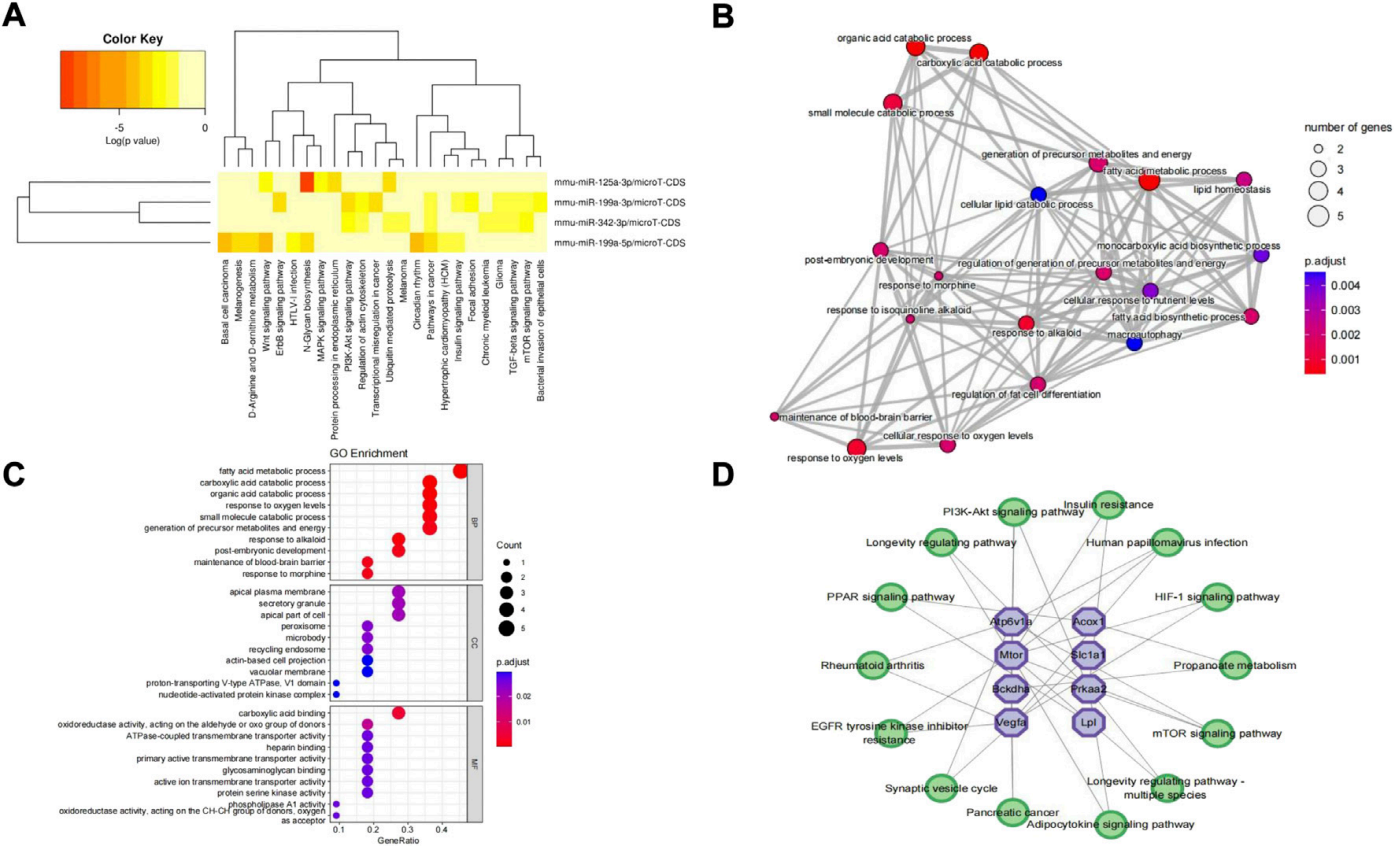
KEGG pathway analysis of DEmiRNAs, GO enrichment analysis of hub genes, and hub gene–KEGG interaction network. **(A)** KEGG pathway analysis of 4 DEmiRNAs obtained from miRPath V3.0. **(B)** Top 20 GO terms of hub genes. The circles represent GO terms. **(C)** Top 10 terms of BP, CC, and MF of hub genes. **(D)** Hub gene–KEGG interaction network. No pathways matched “Pi4ka,” “Myo5a,” and “Aldh5a1” hub genes. Purple octagons indicate hub genes, while green circles indicate pathways. KEGG, Kyoto Encyclopedia of Genes and Genomes; DEmiRNAs, differentially expressed miRNAs; GO, Gene Ontology; BP, biological process; CC, cellular component; MF, molecular function.

In addition, significant results of the KEGG pathway enrichment analysis of 4 validated DEmiRNAs showed that these DEmiRNAs were significantly enriched in the Wnt signaling pathway, phosphatidylinositol 3-kinase (PI3K)-protein kinase B (AKT) signaling pathway, ubiquitin-mediated proteolysis, protein processing in the endoplasmic reticulum, mammalian/mechanistic target of the rapamycin (mTOR) signaling pathway, focal adhesion, transforming growth factor-beta (TGF-beta) signaling pathway, mitogen-activated protein kinase (MAPK) signaling pathway, *etc.* (Figure 7A). In addition, GO terms of the hub genes were markedly enriched in metabolic processes, response to oxygen levels, metabolites and energy, regulation of fat cell differentiation, macroautophagy, *etc.* (Figure 7B). The significant top 10 BP, CC, and MF terms of GO are shown in Figure 7C. In the hub gene–KEGG interaction network, the hub genes were primarily involved in the pathway of propanoate metabolism, mTOR signaling pathway, adipocytokine signaling pathway, epidermal growth factor receptor (EGFR) tyrosine kinase inhibitor resistance, peroxisome proliferator-activated receptor (PPAR) signaling pathway, PI3K-Akt signaling pathway, hypoxia-inducible factor type 1 (HIF-1) signaling pathway, *etc. Pi4ka*, *Myo5a*, and *Aldh5a1* were linked with no pathways and were excluded from the network (Figure 7D).

### Verification of hub genes in humans, construction of the miRNA–mRNA–TF regulatory network across mice and humans, and diagnostic value of key hub genes

**FIGURE 8:**
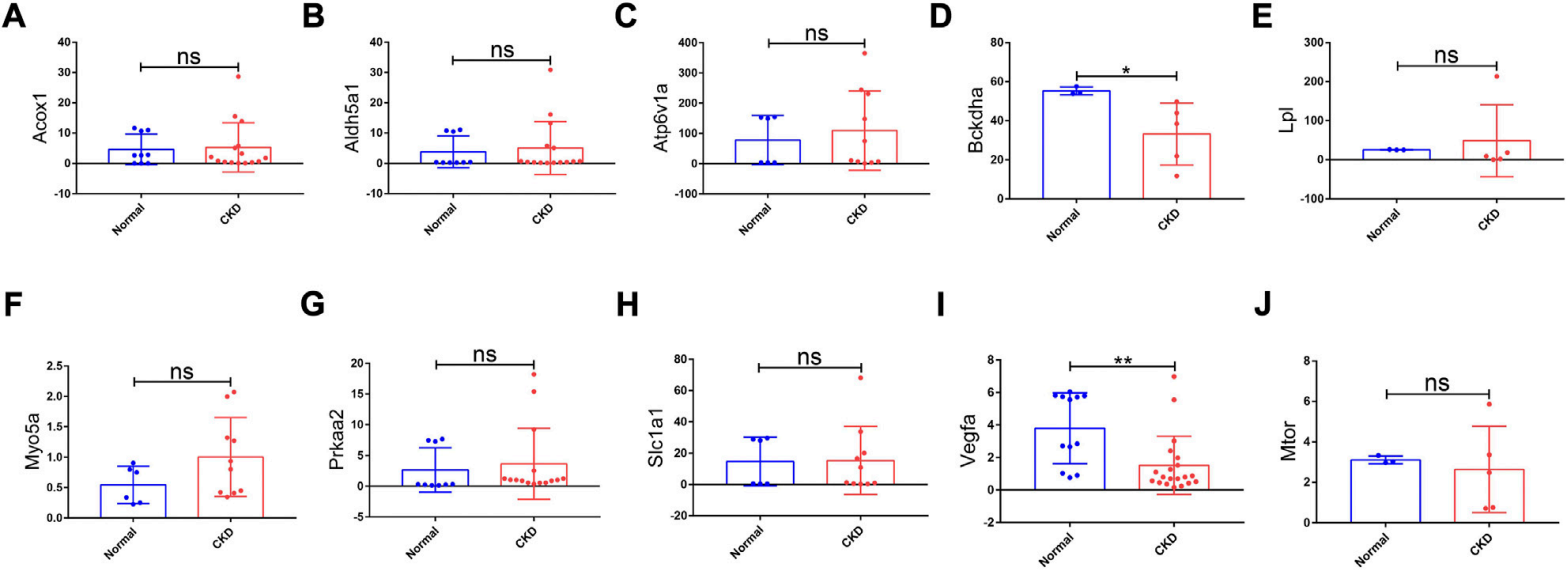
Expression levels of hub genes in CKD kidney tissues and normal kidney tissues based on the validation set of the GSE66494 dataset. **(A)** *Acox1*; **(B)** *Aldh5a1*; **(C)** *Atp6v1a*; **(D)** *Bckdha*; **(E)** *Lpl*; **(F)** *Myo5a*; **(G)** *Prkaa2*; **(H)** *Slc1a1*; **(I)** *Vegfa*; and **(J)** *Mtor*. No results matched “Pi4ka” in the validation set of the GSE66494 dataset. CKD, chronic kidney disease; ns, no significance; *p < 0.05 and **p < 0.01.

**FIGURE 9:**
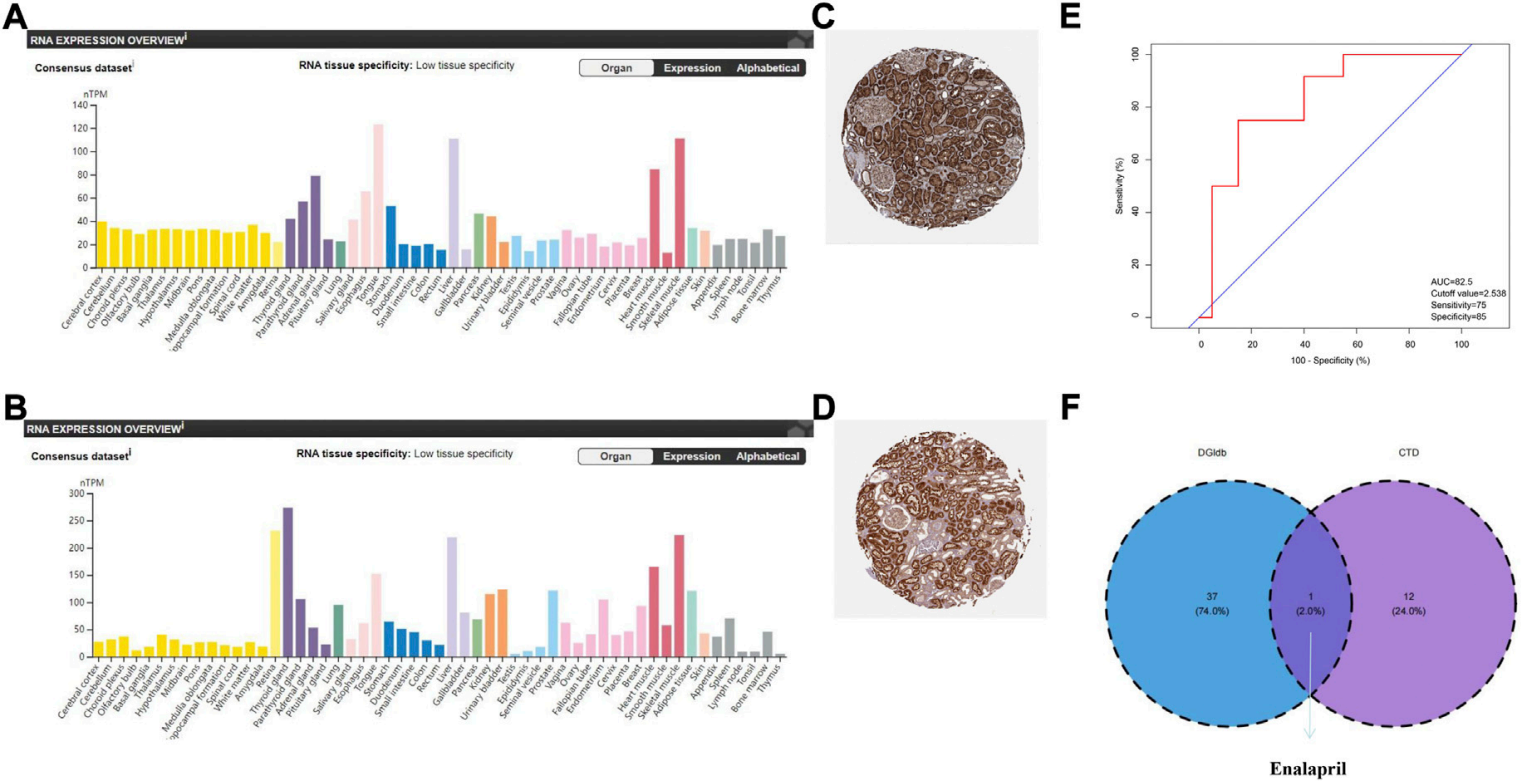
Verification of the expression of key hub genes in humans, diagnostic value of *Vegfa*, and prediction of potential drugs. **(A)** Expression of *Bckdha* in normal kidney tissues. The orange module showed that *Bckdha* was moderately expressed in normal kidneys. **(B)** Expression of *Vegfa* in normal kidney tissues. The orange module showed that *Vegfa* was moderately expressed in normal kidney. **(C)** Immunohistochemical staining showed that *Bckdha* was highly expressed in normal kidney tissues. **(D)** Immunohistochemical staining showed that *Vegfa* was highly expressed in normal kidney tissues. **(A–D)** Diagrams were downloaded from the Human Protein Atlas, available online (http://www.proteinatlas.org). **(E)** Diagnostic value of *Vegfa* in the validation set of the GSE66494 dataset. **(F)** Venn diagram of drug prediction results of *Vegfa*. AUC, area under the curve; DGIdb, Drug–Gene Interaction database; CTD, Comparative Toxicogenomics Database.

To explore the value of these hub genes in clinical practice, the validation set of the GSE66494 dataset was used to validate the expression levels of the hub genes. Of the 11 hub genes, 8 exhibited no significant difference, and *Pi4ka* was not detected. Only *Bckdha* and *Vegfa* were verified in the human dataset and considered key hub genes (Figure 8). In addition, HPA, an online database (http://www.proteinatlas.org), showed that *Bckdha* and *Vegfa* were moderately expressed in normal human kidney tissues (Figures 9A,B). Immunohistochemical staining indicated that *Bckdha* and *Vegfa* were highly expressed in normal kidney tissues (Figures 9C,D). Moreover, in humans, as in mice, *Bckdha* was also predicted by miR-125a-3p *via* miRDB and TargetScan databases. Likewise, *Vegfa* was predicted by miR-199a-5p in humans as well using starBase and TargetScan databases. The sequences of miR-125a-3p and miR-199a-5p in mice were identical to those in humans (Table 2). Subsequently, 6 TFs were co-predicted to regulate *Bckdha* and *Vegfa* across mice and humans, namely, Pax2, Pax5, Sp1, Sp2, Sp3, and Sp4.

**TABLE 2:** Sequences of miR-125a-3p and miR-199a-5p in mice and humans by miRBase.

Accession	ID	Mature accession	Mature ID	Mature sequence
MI0000151	mmu-mir-125a	MIMAT0004528	mmu-miR-125a-3p	ACA​GGU​GAG​GUU​CUU​GGG​AGC​C
MI0000469	hsa-mir-125a	MIMAT0004602	hsa-miR-125a-3p	ACA​GGU​GAG​GUU​CUU​GGG​AGC​C
MI0000241	mmu-mir-199a-1	MIMAT0000229	mmu-miR-199a-5p	CCC​AGU​GUU​CAG​ACU​ACC​UGU​UC
MI0000242	hsa-mir-199a-1	MIMAT0000231	hsa-miR-199a-5p	CCC​AGU​GUU​CAG​ACU​ACC​UGU​UC
MI0000713	mmu-mir-199a-2	MIMAT0000229	mmu-miR-199a-5p	CCC​AGU​GUU​CAG​ACU​ACC​UGU​UC
MI0000281	hsa-mir-199a-2	MIMAT0000231	hsa-miR-199a-5p	CCC​AGU​GUU​CAG​ACU​ACC​UGU​UC

Based on the results of miRNA–mRNA and mRNA–TF pairs, a miRNA–mRNA–TF regulatory network across mice and humans was established, involving 2 key hub genes, 2 miRNAs, and 6 TFs (Figure 5F). In such a regulatory network, miR-125a-3p targeted *Bckdha* and Pax2, Pax5, Sp1, Sp2, Sp3, and Sp4 were the TFs of *Bckdha*, while miR-199a-5p targeted *Vegfa* and Pax2, Pax5, Sp1, Sp2, Sp3, and Sp4 were the TFs of *Vegfa*.

Next, an ROC curve analysis was conducted on *Bckdha* and *Vegfa* in the validation set of the GSE66494 dataset and the area under the curve (AUC) value of *Vegfa* was 0.825 (*p* = 0.002) (Figure 9E). Due to too little test data, the qualified ROC curve on *Bckdha* was not available.

### Prediction of potential drugs

Nephrosclerosis was inserted into the CTD, and then 1 and 13 potential chemicals that could affect the expression levels of *Bckdha* and *Vegfa* were predicted, respectively. In addition, using DGIdb, 38 drugs were selected as potential drugs targeting *Vegfa*, while no drug was observed to target *Bckdha*. Finally, one drug targeting *Vegfa,* enalapril, co-appeared in the CTD and DGIdb (Table 3, Figure 9F).

**TABLE 3:** Drugs that can target *Vegfa* and *Bckdha* as predicted by the CTD and DGIdb.

Database	Gene name	Disease	Drugs
CTD	*Vegfa*	Nephrosclerosis	Benazepril, dibutyl phthalate, dietary fats, doxorubicin, enalapril, honokiol, hydrocortisone, ibuprofen, losartan, metformin, niclosamide, streptozocin, valsartan
	*Bckdha*	Nephrosclerosis	Streptozocin
DGIdb	*Vegfa*	n/a	Ranibizumab, bevasiranib, pegaptanib sodium, Elmiron, aflibercept, risuteganib, brolucizumab, bevacizumab-In^111^, bevacizumab, MP-0250, CDC-801, navicixizumab, Zaltrap, abicipar pegol, conbercept, squalamine, sildenafil, domatinostat, cilostazol, oxaliplatin, leucovorin, enalapril, muparfostat, gentamicin, capecitabine, lenalidomide, fenofibrate, irinotecan, phenytoin, docetaxel, sunitinib, fluorouracil, sorafenib, regorafenib, celecoxib, carboplatin, OSI-632, cisplatin
	*Bckdha*	n/a	NO

CTD, Comparative Toxicogenomics Database; DGIdb, Drug–Gene Interaction database.

## Discussion

Although the causes of CKD are varied and complex, RF is the common final pathological phenotype of all CKD (Boor et al., 2010). A comprehensive understanding of the molecules and pathways involved in RF will provide potential therapeutic targets and improve the survival and prognosis of patients with RF. In the present study, 4 DEmiRNAs and 11 hub genes were identified in the miRNA–mRNA regulatory network in mice by integrated analysis of five mouse 7dUUO datasets, and the roles of some of them have been reported in the pathogenesis of tissue fibrosis or RF. Many studies have demonstrated the importance of upregulated miR-199a-3p in mouse cardiac fibrosis (Liang et al., 2018; Zeng et al., 2021) and rat liver fibrosis (Yang et al., 2020a). Consistently, miR-199a-3p is upregulated in human myocardium with cardiac remodeling (Zeng et al., 2021), and miR-199a-3p liver expression was increased in patients with cirrhosis (Yang et al., 2020a). Kidney biopsies from patients with diabetic nephropathy (DN) and IgA nephropathy exhibited a substantial increase in profibrotic proteins and miR-199a-3p (Yang et al., 2017). Similarly, miR-199a-5p has been reported to participate in various kinds of tissue fibrosis, such as peritoneal fibrosis (Che et al., 2017), cardiac fibrosis (Zeng et al., 2021), lung fibrosis (Yang et al., 2022), and liver fibrosis (Broermann et al., 2020; Ding et al., 2021; Messner et al., 2021), in animal and cell studies. Moreover, miR-199a-5p is also upregulated in the human myocardium with cardiac remodeling (Zeng et al., 2021). Additionally, the expression of miR-199a-5p was significantly enhanced in the lungs (Lino et al., 2013) and serum (Yang et al., 2015; Shi et al., 2021) of idiopathic pulmonary fibrosis (IPF) patients and in liver biopsy specimens of patients with hepatic fibrosis (Murakami et al., 2011). Lino et al. demonstrated that the expression of miR-199a-5p was increased in the UUO model of RF (Lino et al., 2013). The expression of miR-199a-5p was also dramatically elevated in human autosomal dominant polycystic kidney disease tissues (Sun et al., 2015) and in IgA nephropathy patients with interstitial and glomerular fibrotic lesions (Hennino et al., 2016). Inconsistent with our results, miR-125a-3p inhibited RF by downregulating transforming growth factor (TGF)-β1 in lupus nephritis (Zhang et al., 2019) and the overexpression of miR-342-3p suppressed high glucose-induced renal interstitial fibrosis in DN (Jiang et al., 2020). These different results may be due to discrepancy in stimuli, cell lines, animal models, and species. On the other hand, some of the KEGG pathways of the 4 DEmiRNAs were matched with the pathogenesis of the RF reported. The Wnt signaling pathway is involved in tissue fibrosis in various diseases (Hu et al., 2020; Pulkkinen et al., 2008), including kidney fibrosis in the course of acute kidney injury, chronic kidney disease, chronic kidney disease-associated vascular injury and mineral bone disease, and cystic kidney disease (Schunk et al., 2021). The PI3K-AKT signaling pathway plays crucial role in the pathogenesis of RF by regulating the degradation of the extracellular matrix and epithelial cell–mesenchymal transition of kidney tubular epithelial cells (Meng et al., 2016; Hu et al., 2021). Cell growth, proliferation, autophagy, epithelial–mesenchymal transition, and endoplasmic reticulum stress which are tightly associated with RF were regulated by the mTOR signaling pathway (Hu et al., 2015; Fantus et al., 2016; Liu et al., 2020), and some small molecules attenuate kidney interstitial fibrosis by repressing kidney myofibroblast activation through the mTOR signaling pathway (Feng et al., 2021). Focal adhesion signaling plays an important role in cyst growth and fibrosis in polycystic kidney disease (Zhang et al., 2020). The TGF-β signaling pathway is crucial in RF, and during fibrotic progression, complex regulatory networks composed of multiple TGF-β-related signaling pathways work together (Yang et al., 2016; Finnson et al., 2020). Increased PAR2 expression and the MAPK signaling pathway contribute to RF by increasing the inflammatory responses and promoting the epithelial–mesenchymal transition process (Ha et al., 2022). CYP4A14 is responsible for RF caused by AngII in mice, also mainly by activating the MAPK signaling pathway (Zhou et al., 2018). In addition, antagonizing the MAPK signaling pathway alleviated epithelial–mesenchymal transition and RF (Li et al., 2017; Deng et al., 2020). Collectively, although not entirely consistent with our analysis results, the aforementioned studies indicated that these 4 DEmiRNAs might participate in the pathogenesis of RF and might be potential novel targets for RF treatment; however, more experimental evidence is required.

In addition to the 4 DEmiRNAs, some of the hub genes in the miRNA–mRNA regulatory network have been reported in organ fibrosis. Xie et al. observed that the *Acox1* expression level was lower in UUO RF mice than in non-UUO (Xie et al., 2021). Kidneys from cats with CKD had significantly lower *Vegfa* levels than those from healthy control cats, and *Vegfa* levels were negatively connected to histologic score severity (Lourenço et al., 2020). Baelde et al. found a decline in *Vegfa* expression in renal biopsies of patients with DN compared with control kidneys (native kidneys with normal histology and function, cadaver donor kidneys unfit for transplantation because of technical reasons, and the unaffected part of tumor nephrectomy samples); moreover, there was a relationship between the decrease in glomerular *Vegfa* levels and the amount of interstitial fibrosis (Baelde et al., 2007). In addition to DN, reduced *Vegfa* levels were connected to enhanced glomerular histological activity and poor kidney prognosis in patients with lupus nephritis (Avihingsanon et al., 2009). Moreover, a *Vegfa* supplement could be used for DN therapy, as *Vegfa* could decrease interstitial HIF1A to attenuate tubulointerstitial fibrosis (Li et al., 2020). *Vegfa* treatment induced a transfer in macrophage infiltration into the renal tissues from the injurious M1 phenotype to the anti-inflammatory M2 phenotype, raising the probability that *Vegfa* treatment may directly inhibit kidney inflammation and subsequent fibrosis (Tanabe et al., 2020). Nevertheless, increased kidney tubular *Vegfa* expression has also been shown to cause fibrosis and glomerular disease (Veron et al., 2014; Kikuchi et al., 2019). Lopes et al. (2019) observed that in patients with glomerular diseases, the renal *Vegfa* level was correlated positively with the extent of RF. The opposite result may be due to 72% of biopsies in Lopes et al.’s research being conducted in the more early stages of glomerular and tubulointerstitial damage. These data indicate that *Acox1* and *Vegfa* may be important molecular targets in the pathogenesis of RF. Nevertheless, some of the hub genes in our miRNA–mRNA network were poorly studied and have not been reported in RF before. The expression changes of *Slc1a1*, *Myo5a*, and *Aldh5a1* were known to affect cancer cell proliferation migration, invasion, and metastasis and may be critical for carcinoma pathophysiology (Tian et al., 2017; Ergün et al., 2019; Tomic et al., 2020; Deng et al., 2021). In addition, a previous study revealed the correlation between the loss of *Aldh5a1* and the presence of epithelial–mesenchymal transition (Hilvo et al., 2016). *Bckdha* has been reported to play a protective role in liver fibrosis (Wang et al., 2012). Conversely, Yang et al. suggested that pyridostigmine downregulated p-*Bckdha*/*Bckdha* to improve cardiac branched-chain amino acid catabolism and alleviated cardiac hypertrophy and fibrosis in diabetic cardiomyopathy mice (Yang et al., 2021). The expression of *Lpl* contributed to hepatic fibrosis in nonalcoholic steatohepatitis (Teratani et al., 2019). In addition, some of the functional and pathway enrichment analyses of the hub genes were also matched with the pathogenesis of RF reported. In recent years, increasing evidence has demonstrated that energy metabolism plays a major regulatory role in the progression of RF (Lan et al., 2016; Liu et al., 2017). Autophagy dysfunction is implicated in the pathogenesis of acute kidney injury and RF diseases (Nam et al., 2019; Yang et al., 2020b; Tang et al., 2021). Existing research further indicates that in RF, the process of autophagy may either promote it by contributing to tubular atrophy and decomposition or prevent it by improving intracellular degradation of excessive type I collagen mediated by TGF-β1 (He et al., 2014). In addition, another study demonstrated the vital role of autophagy in endothelial cell integrity, and the disruption of endothelial autophagy could give rise to significant pathological interleukin 6-dependent endothelial-to-mesenchymal transition and organ fibrosis (Takagaki et al., 2020). In tumor genesis, propanoate metabolism plays a part in lipogenesis, *via* downregulating propanoate metabolism and lipogenesis, resulting in the suppression of angiogenesis *in vitro*/*in vivo* (Hussain et al., 2016; Huang et al., 2020). As mentioned previously, the mTOR signaling pathway and PI3K-AKT signaling pathway are tightly connected with RF. A study on the process of DN progression found that the adipocytokine signaling pathway is uniquely enriched in end-stage renal disease (Wang et al., 2019). The hypoxia-inducible factor 1(HIF-1) signaling pathway is a pivotal pathway in renal injury and fibrosis (Hasan et al., 2017; Kabei et al., 2018). Therefore, it is speculated that these hub genes may be involved in the molecular mechanism of RF, which is worthy of further study.

In the present study, we observed that the expressions of key hub genes (*Bckdha* and *Vegfa*) were significantly downregulated in CKD tissues, which was highly consistent with the results in mice, indicating their importance in human RF and suggesting that these key hub genes have great research value in humans. Moreover, *Bckdha* and *Vegfa* were moderately expressed in normal human kidney tissues and immunohistochemical staining showed that *Bckdha* and *Vegfa* were highly expressed in normal kidney tissues (data from the HPA, an online database (http://www.proteinatlas.org)), which suggest their importance in maintaining normal physiological function. The decrease in renal *Vegfa* expression was related to a reduction in peritubular capillary density (Lindenmeyer et al., 2007), while capillary rarefaction would reduce the supplement of oxygen and blood; this results in tubular cell viability loss, tubular atrophy, and interstitial fibrosis (Afsar et al., 2018), which supported the findings that the decrease in kidney vessels, cortical atrophy, and urinary protein was further exacerbated in patients with reduced expression of *Vegfa* (Lindenmeyer et al., 2007). In the hub gene–KEGG interaction network, *Bckdha* was enriched in propanoate metabolism which was involved in tumor genesis, and the role in RF has not been studied before. In addition, *Vegfa* was enriched in the PI3K-AKT signaling pathway and HIF-1 signaling pathway, which were closely associated with RF. Fascinatingly, exogenous expression of CD147 in hepatocytes contributed to the expression and secretion of *Vegfa* through the PI3K/AKT pathway in liver fibrosis (Yang et al., 2015). Presumably, these key hub genes and related pathways might be involved in the occurrence and development of RF, which is of great significance for further research. To our excitement, as predicted in mice, *Bckdha* and *Vegfa* were also predicted by miR-125a-3p and miR-199a-5p, respectively, in humans by online predicted databases. Furthermore, to further verify miRNA conservation between species, sequence analysis was performed and showed that the sequences of miR-125a-3p and miR-199a-5p in mice were identical to those in humans. Strikingly, in patients with IPF, upregulated miR-199a-5p regulated *Vegfa* and led to the development of IPF or other fibrosis-related diseases (Zhu et al., 2021). Of course, we noticed that many hub genes have no significance in human samples, which may be caused by the differences between species. Although humans and mice had very high genetic homology, different species may lead to some bias in results. In addition, some gene expansions might be greatly temporally and spatially specific, which resulted in discordance.

Subsequently, 6 TFs, namely, Pax2, Pax5, Sp1, Sp2, Sp3, and Sp4, were co-predicted to regulate *Bckdha* and *Vegfa* across mice and humans. Some of them also have been reported in RF. Pax2 promotes epithelial–mesenchymal transition in renal tubular epithelia and, thus, plays a crucial part in RF (Hou et al., 2018). Sp1 is an essential transcriptional regulator for the antifibrotic protein follistatin and was identified as a profibrotic factor in kidney disease (Mehta et al., 2019). Sp1 has been shown to be a mediator of fibrosis in renal tubular epithelial cells through the activation of type 1 collagen expression during the mesenchymal transition (Tian et al., 2021). Nevertheless, Sp1 also regulates concurrent protective responses to restrain the extent of fibrosis, which could mediate the induction of antifibrotic protein Smad7 *via* the transforming growth factor-beta (Brodin et al., 2000). The different results are potentially on account of discrepancy in the regulation of pathways in different microenvironments and cells. Sp3 played a major role in human corneal endothelium fibrosis (Lee et al., 2020). In addition, Pax5 was aberrantly expressed in tumors (Benzina et al., 2017; Dong et al., 2018) and human skin fibrosis (Song et al., 2018; Qin et al., 2021). These TFs might be important factors in the pathogenesis of RF. While Sp2, Sp3, and Sp4 were associated with cell proliferation, survival, migration, and invasion and highly expressed in tumors (Zhao et al., 2014; Kim et al., 2017; Safe et al., 2021), their roles in fibrosis have yet to be reported. Whether and how these TFs participate in the pathogenesis of RF remain to be further studied.

Next, ROC curve analysis was performed on *Bckdha* and *Vegfa* and excavated *Vegfa* with an AUC value over 0.80, illustrating that *Vegfa* exhibited a good differentiating ability to discriminate CKD samples from normal samples and could be used as a marker for the diagnosis of RF. Consistently, Rudnicki et al. also proposed that *Vegfa* expression was repressed in patients with progressive renal failure and was negatively associated with the degree of interstitial fibrosis. *Vegfa* was significantly superior in predicting clinical outcome as compared to the degree of tubular atrophy and interstitial fibrosis at the time of biopsy (Rudnicki et al., 2009). Unfortunately, it is difficult to evaluate the diagnostic value of *Bckdha* due to too little testing data.

In addition to helping uncover potential disease mechanisms, identifying the underling regulatory mechanism responsible for gene expression changes under disease conditions could assist in enabling the discovery of drug targets. Enalapril targeting *Vegfa* was co-predicted by the CTD and DGIdb. Enalapril may exert antifibrotic effects on a variety of fibrosis tissues, such as peritoneal fibrosis (Lee et al., 2011), pathological cardiac hypertrophy and fibrosis (Kushwaha et al., 2012; Ham et al., 2018), liver fibrosis (Kushwaha et al., 2012), and kidney fibrosis (Kushwaha et al., 2012; Molnar et al., 2018), even if the short-term use of enalapril confers long-term protection against target organ damage (Hale et al., 2012), or delayed intervention with enalapril had parallel effects on tubulointerstitial, vascular damage, and glomerulosclerosis, with regression of existing lesions (Adamczak et al., 2003). Enalapril significantly alleviates mesangial matrix expansion, interstitial collagen IV deposition, and renal fibrosis (Ougaard et al., 2018; Greite et al., 2020; Veitch et al., 2021). Enalapril also attenuated renal fibrosis in UUO rats by suppressing apoptosis of renal tubular epithelial cells (Yang et al., 2019) or regulating fibroblast activation (ɑ-SMA), pro-inflammatory cytokine TGF-β, mast cell infiltration, and, probably, mast cell degranulation (Sun et al., 2016). Moreover, in the study by Kee Hwan, the angiogenic response could initially occur because of increased survival from VEGF-A/VEGF receptor-1 signaling in response to kidney damage by enalapril (Yoo et al., 2018). Consistently, the VEGF-A protein expression level was significantly enhanced in enalapril-treated rat kidneys (Yim et al., 2016). In addition, as mentioned previously, capillary rarefaction would decrease the supplement of oxygen and blood, leading to interstitial fibrosis, while the amelioration of rarefaction by enalapril treatment indicates an ethological role of rarefaction dependent on hypoxia in the progression of fibrosis (Hamar and Kerjaschki, 2016). More to the point, in the nephrotoxic serum nephritis model, enalapril reversed the regulation of several inflammation and fibrosis-associated genes, which were also regulated in CKD patients (Ougaard et al., 2020). These studies suggest that enalapril might act as a potential direction for future treatment in RF, although its exact mechanism in RF still requires further investigation. Notably, although enalapril was indicated to be a promising method to combat fibrosis, additional clinical studies are needed to confirm effectiveness and long-term safety in patients at various stages of RF.

Herein, some measures have been taken to ensure the reliability of the study. For instance, the model condition of RF from different datasets is the same. Independent datasets were applied to validate the DEmiRNAs and mRNAs targeted by DEmiRNAs in the present study. Despite the aforementioned strengths, there are still some limitations. First, DEmiRNA and DEmRNA analysis would inevitably filter out some important miRNAs and mRNAs with minor changes. Second, the sample size of the datasets utilized for analysis obtained from the GEO is relatively small and it was difficult to exclude potential random errors and false positives. Third, molecular biology experimental validation of the results was not performed because of resource limitations. Therefore, larger datasets as well as experimental and clinical studies are needed to validate these results.

In summary, our study initially constructed the miRNA–mRNA–TF network, and Pax2, Pax5, Sp1, Sp2, Sp3, and Sp4 together with *Bckdha*-dependent miR-125a-3p/*Vegfa*-dependent miR-199a-5p formed a co-regulatory network, enabling *Bckdha*/*Vegfa* to be tightly controlled in the underlying pathogenesis of RF. In addition, *Vegfa* showed good diagnostic value, which might be used as a potential novel diagnostic marker and may be targeted by enalapril for RF therapy. These will further the understanding of the molecular mechanism of RF and may provide a novel research reference orientation for further in-depth research studies on the mechanism of action, diagnosis, prevention, and personalized therapeutic targets for RF.

## Data Availability

Original datasets are available in a publicly accessible repository. The original contributions presented in the study are publicly available. This data can be found here: [https://www.ncbi.nlm.nih.gov/geo/]. The data presented in the study are deposited in the Gene Expression Omnibus (GEO) database repository, accession number: GSE118340, GSE162794, GSE42716, GSE85209, GSE118339, GSE66494.

## Glossary

7dUUO: 7 days of UUO
ACE: angiotensin-converting enzyme
AKT: protein kinase B
AUC: area under the curve
BP: biological process
CC: cellular component
CKD: chronic kidney disease
CTD: Comparative Toxicogenomics Database
DC: degree centrality
DEmiRNAs: differentially expressed miRNAs
DEmRNAs: differentially expressed mRNAs
DGIdb: drug–gene interaction database
DN: diabetic nephropathy
ESRD: end-stage renal disease
GEO: Gene Expression Omnibus
GO: Gene Ontology
HIF: hypoxia-inducible factor
HPA: Human Protein Atlas
IPF: idiopathic pulmonary fibrosis
KEGG: Kyoto Encyclopedia of Genes and Genomes
LogFC: log fold change
MAPK: mitogen-activated protein kinase
MF: molecular function
miRNA: microRNA
mRNA: messenger RNA
mTOR: mammalian/mechanistic target of rapamycin
NCBI: National Center for Biotechnology Information
NES: normalized enrichment score
PCA: principal component analysis
PI3K: phosphatidylinositol 3-kinase
PPI: protein–protein interaction
RF: renal fibrosis
ROC: receiver operating characteristic
SPSS: statistical product and service solutions
STRING: Search Tool for the Retrieval of Interacting Genes/Proteins
TF: transcription factor
TGF: transforming growth factor
UUO: unilateral ureteral obstruction
