# Supplementary figures and images for: Comprehensive analyses of the microRNA–messenger RNA–transcription factor regulatory network in mouse and human renal fibrosis
Source: Front Genet. 2022 Nov 15;13:925097. doi: 10.3389/fgene.2022.925097 (PMC9705735; doi:10.3389/fgene.2022.925097)

A

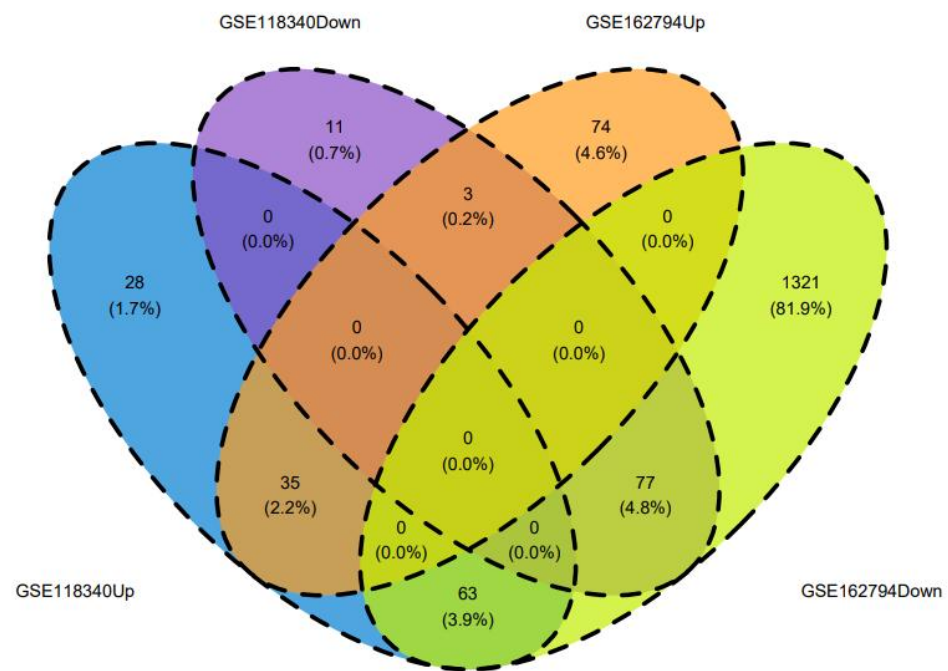

B

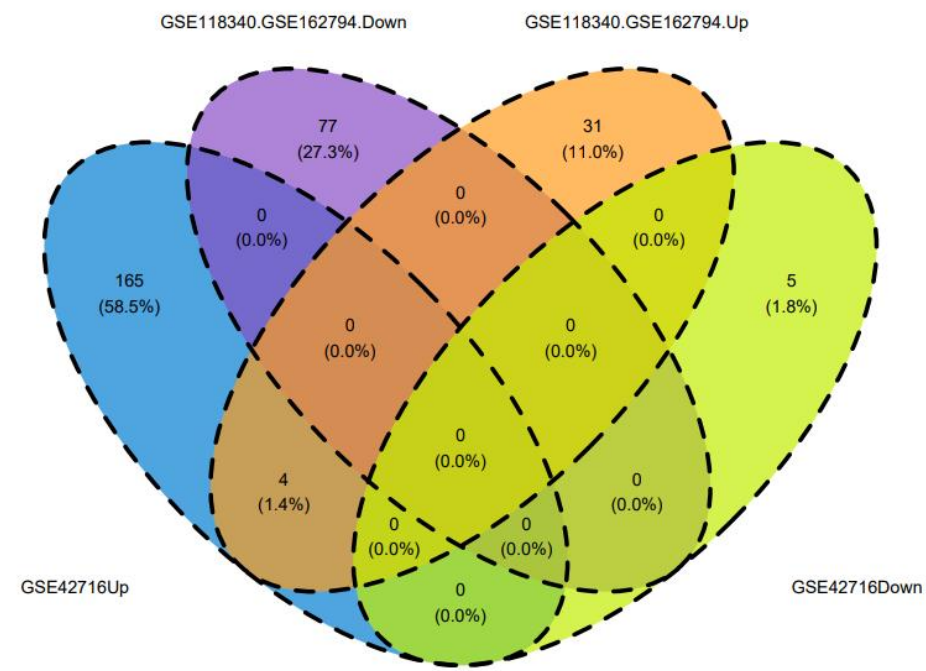

Supplement: Supplementary file 1 [file DataSheet2.pdf]

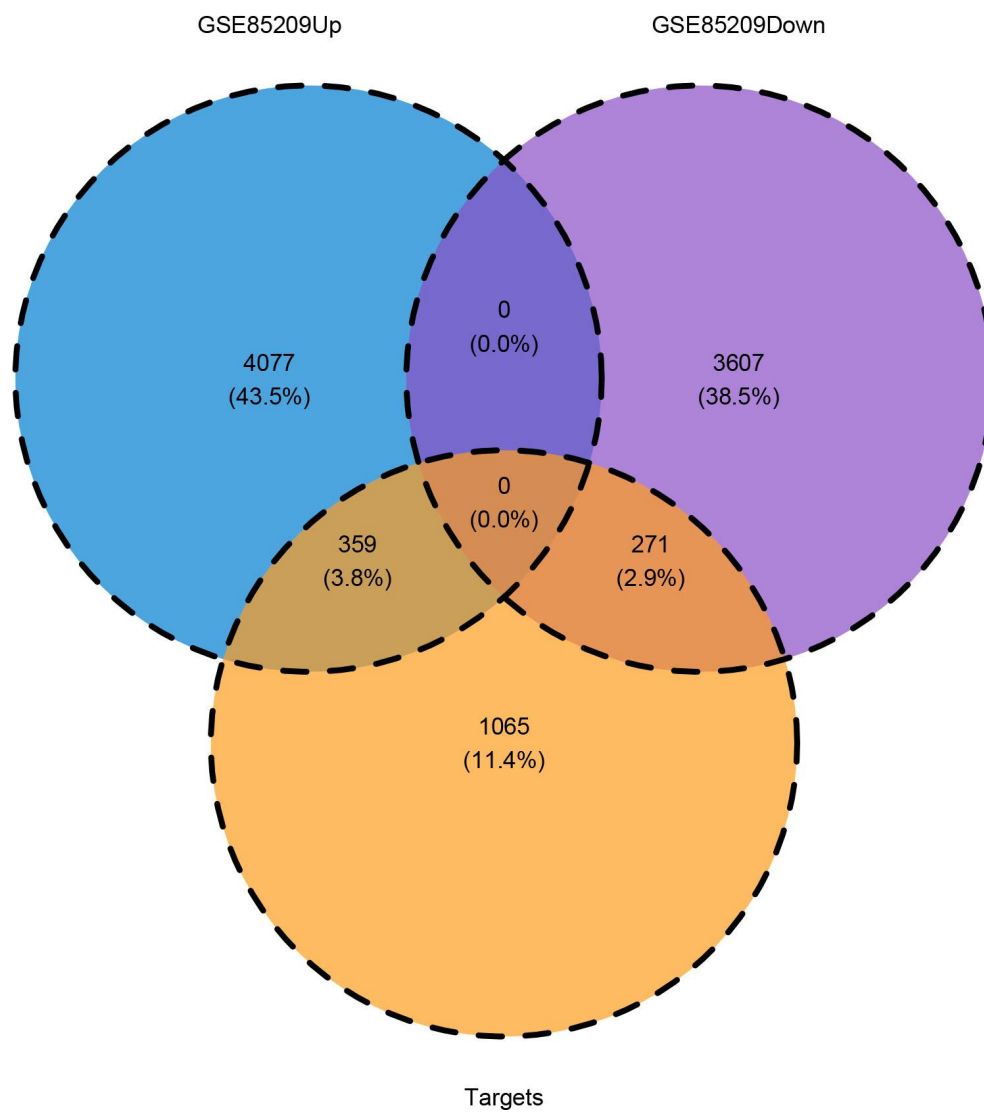

Supplement: Supplementary file 2 [file DataSheet4.pdf]

# A

Individuals - PCA

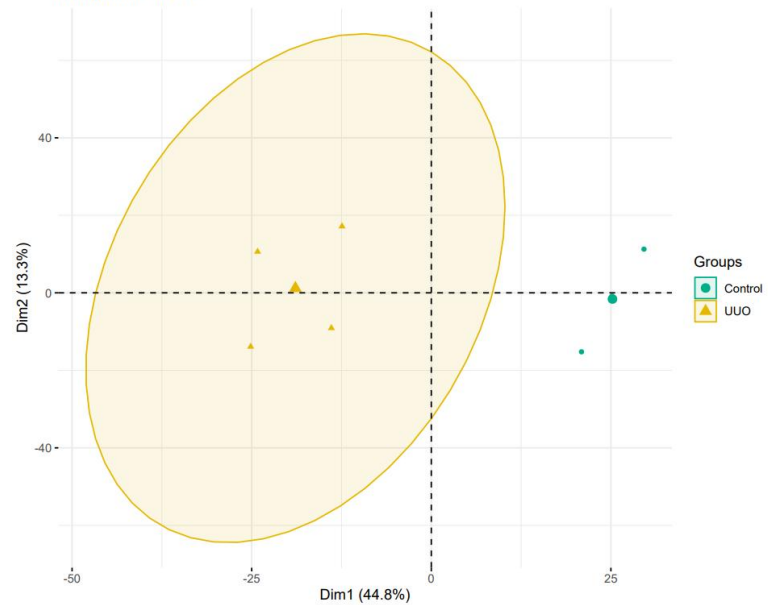

# B

Individuals - PCA

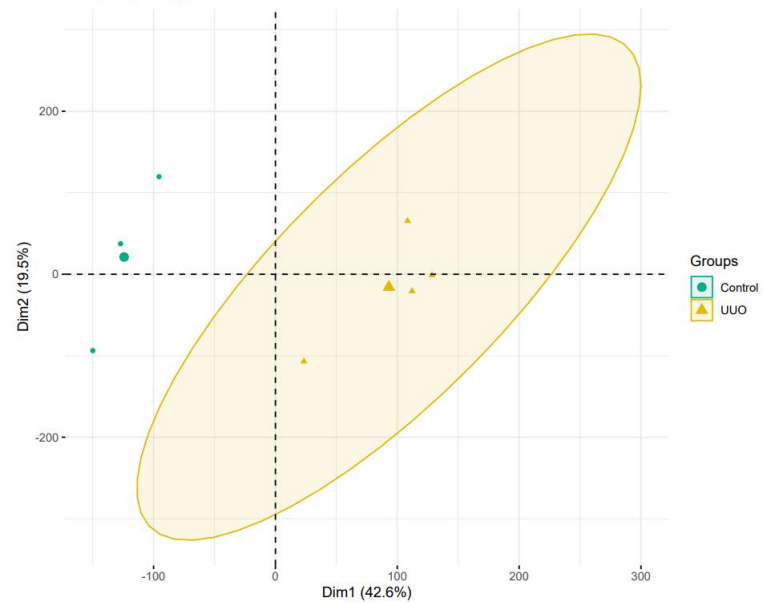

# C

Individuals - PCA

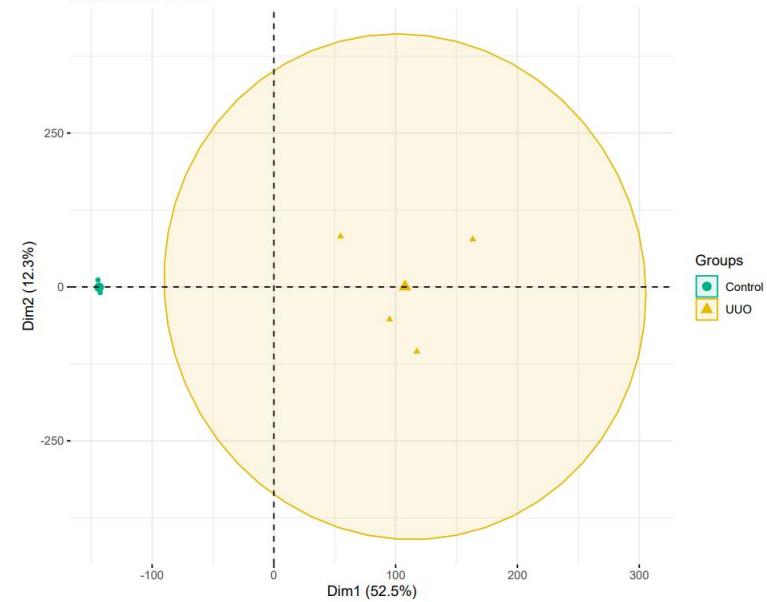

Supplement: Supplementary file 5 [file DataSheet1.pdf]

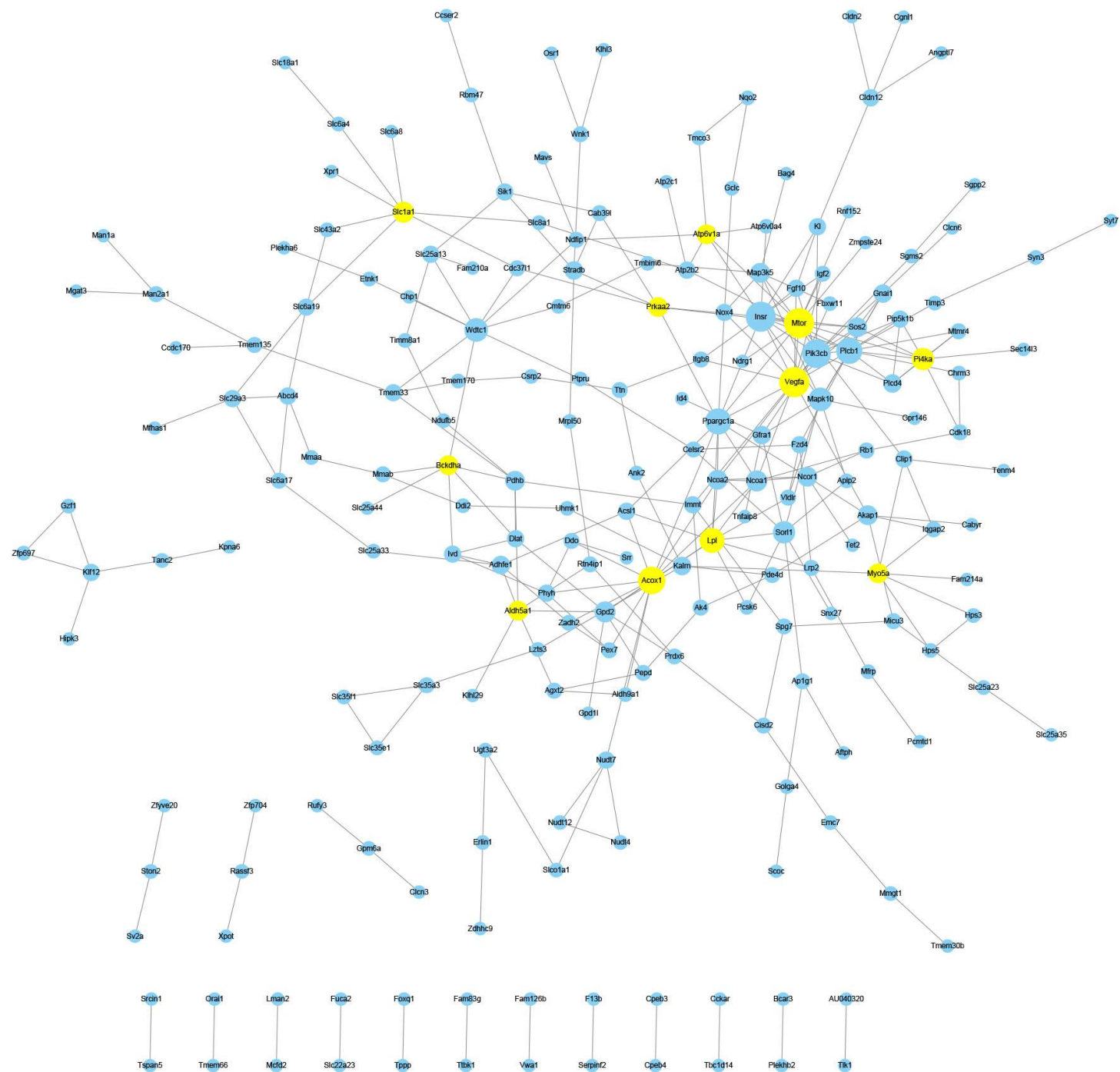

Supplement: Supplementary file 6 [file DataSheet5.pdf]
