# Supplementary material for: Comprehensive analyses of the microRNA–messenger RNA–transcription factor regulatory network in mouse and human renal fibrosis
Source: Front Genet. 2022 Nov 15;13:925097. doi: 10.3389/fgene.2022.925097 (PMC9705735; doi:10.3389/fgene.2022.925097)
Supplement: Supplementary file 3 [file DataSheet3.pdf]

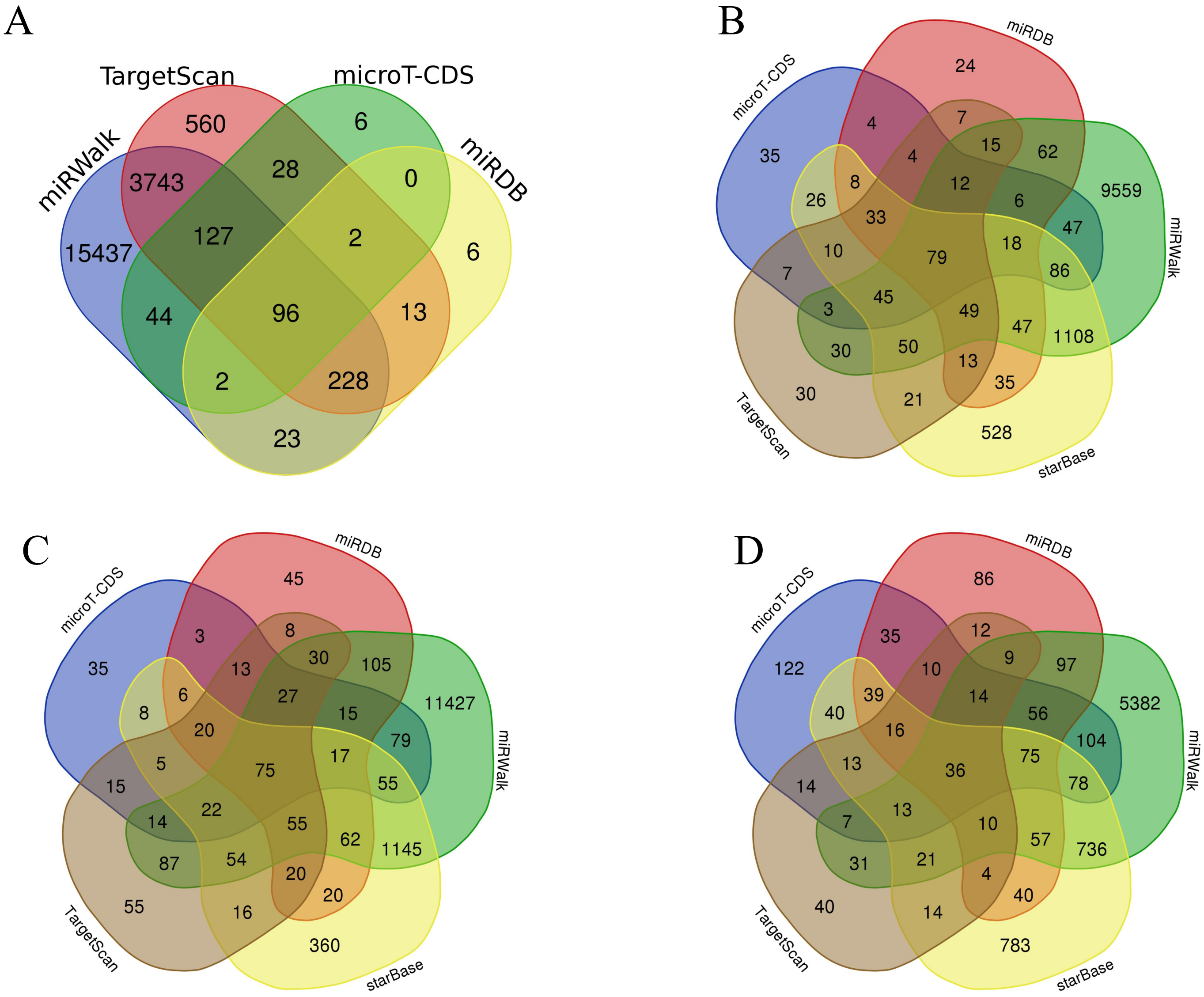

A
TargetScan
microT-CDS
miRWalk
miRDB
560
6
3743
28
0
127
2
15437
6
44
96
13
2
228
23
B
microT-CDS
miRDB
miRWalk
TargetScan
starBase
24
4
7
35
4
15
62
8
12
9559
26
6
33
47
10
79
18
7
86
45
3
49
47
1108
30
50
13
35
30
21
528
C
microT-CDS
miRDB
miRWalk
TargetScan
starBase
45
3
8
35
13
30
105
6
27
11427
8
15
20
79
5
75
17
15
55
22
14
55
62
1145
87
54
20
20
55
16
360
D
microT-CDS
miRDB
miRWalk
TargetScan
starBase
86
35
12
122
10
9
97
39
14
5382
40
56
16
104
13
36
75
14
78
13
7
10
57
736
31
21
4
40
40
14
783
