# Supplementary material for: Comprehensive analyses of the microRNA–messenger RNA–transcription factor regulatory network in mouse and human renal fibrosis
Source: Front Genet. 2022 Nov 15;13:925097. doi: 10.3389/fgene.2022.925097 (PMC9705735; doi:10.3389/fgene.2022.925097)
Supplement: Supplementary file 4 [file Table1.DOC]

**Figure legends**

Supplementary Figure 1. PCA plot and differential expression data in UUO group and control group in three datasets. PCA plot showed the tight clustering of biological replicates and distinct clustering between UUO group and control group in (A) GSE118340, (B) GSE85209 and (C) GSE118339. PCA: principal component analysis; UUO: unilateral ureteral obstruction.

Supplementary Figure 2. Identification of overlapping DEmiRNAs in different datasets. (A) GSE118340 and GSE162794. (B) GSE118340.GSE162794. and GSE42716. DEmiRNAs: differentially expressed miRNAs; GSE118340.GSE162794.: intersection of consistent DEmiRNAs in GSE118340 dataset and GSE162794 dataset.

Supplementary Figure 3. The common target mRNAs of DEmiRNAs were predicted by microT-CDS, miRDB, miRWalk, starBase, TargetScan databases. No results matched “miR-125a-3p” in the starBase database. (A) The target mRNAs of miR-125a-3p. (B) The target mRNAs of miR-199a-3p. (C) The target mRNAs of miR-199a-5p. (D) The target mRNAs of miR-342-3p. DEmiRNAs: differentially expressed miRNAs.

Supplementary Figure 4. Venn diagram showed overlapping mRNAs between predicted target mRNAs and DEmRNAs in GSE85209 dataset. DEmRNAs: differentially expressed mRNAs.

Supplementary Figure 5. PPI network of candidate target mRNAs. The yellow circles represented hub genes. PPI: protein-protein interaction.
