# Supplementary material for: Comprehensive analyses of the microRNA–messenger RNA–transcription factor regulatory network in mouse and human renal fibrosis
Source: Front Genet. 2022 Nov 15;13:925097. doi: 10.3389/fgene.2022.925097 (PMC9705735; doi:10.3389/fgene.2022.925097)
Supplement: Supplementary file 7 [file Table2.DOC]

Supplementary Table 1. The top 26 nodes in the PPI network.

| Node name | Degree | Betweenness | Closeness |
| --- | --- | --- | --- |
| Mtor | 14 | 4930.518 | 0.023849893 |
| Insr | 14 | 3801.9553 | 0.023855286 |
| Vegfa | 14 | 3523.0024 | 0.023763938 |
| Pik3cb | 13 | 3992.5452 | 0.023769291 |
| Acox1 | 12 | 4411.3076 | 0.023678599 |
| Plcb1 | 11 | 1797.6482 | 0.023646755 |
| Ppargc1a | 11 | 4666.6694 | 0.023844503 |
| Lpl | 10 | 2941.5466 | 0.023697214 |
| Wdtc1 | 9 | 3828.876 | 0.02351499 |
| Mapk10 | 9 | 1636.8455 | 0.023580689 |
| Pi4ka | 8 | 757.661 | 0.023501894 |
| Sorl1 | 8 | 2462.2004 | 0.023525476 |
| Slc1a1 | 7 | 2584.3674 | 0.02323021 |
| Ncoa1 | 7 | 778.7395 | 0.023649406 |
| Gpd2 | 7 | 2414.4785 | 0.023559624 |
| Sos2 | 6 | 210.1653 | 0.023614997 |
| Myo5a | 6 | 1684.4159 | 0.02319446 |
| Prkaa2 | 6 | 4514.083 | 0.023790732 |
| Bckdha | 6 | 2104.4949 | 0.023473134 |
| Map3k5 | 6 | 984.89404 | 0.023562256 |
| Atp6v1a | 6 | 1680.6117 | 0.023644106 |
| Pdhb | 6 | 2865.8242 | 0.023551736 |
| Ncor1 | 6 | 870.7956 | 0.023522854 |
| Akap1 | 6 | 2185.3928 | 0.023475746 |
| Aldh5a1 | 6 | 1274.2313 | 0.023423623 |
| Ncoa2 | 6 | 470.94318 | 0.023641456 |
